# From Our Correspondents

**Published:** 1986-04

**Authors:** 


### From Our Correspondents

### Frenchay District

Administrative changes every few years are the norm in the National Health Service and, in spite of them all, the service to patients in the United Kingdom is still one of the best in the world, because of the dedication of those closest to the patient. Admittedly, the service falls short of the ideal in honouring promises to treat people on long waiting lists for 'cold' surgery, and in coping with the elderly and underprivileged. No amount of administrative reshuffling will solve the problems of chronic underfunding, but Griffiths' principles show signs of reducing inefficiences and speeding up decision making. The future now lies in the hands and hearts of a few powerful managers and will depend upon their integrity, energy and strength of moral fibre.

At Frenchay, men of high calibre have been chosen with care. There were last minute changes in the apportionment of managers to units and we eventually settled on two main groupings of units straightaway, rather than four units gradually evolving to two over a number of years. The concern of the District Health Authority to achieve the most effective structure tailored to the candidates available was evident and led to some surprising manoeuvres. The main structures are now established and there is a willingness throughout the district to make them work in the best interests of patients.

Dr Paul Walker was appointed as District General Manager and started work in May 1985. He came from North East Thames District where he had been Senior Medical Officer to that large and complex region. He has already set high standards in the dedication and industry which he applies to his new post and has rapidly become conversant with the diverse activity within the Frenchay District.

The Manager for the General Unit, comprising Frenchay Hospital, Manor Park Hospital, Cossham Hospital and the Burden Neurological Institute, was appointed in October. Mr Derek Smith was well known in Bristol, having been District Planning Officer for the Southmead Group before his post as Senior Administrative Officer at Frenchay. He is known for his breadth of understanding of Medical matters, his energy, his fairness and directness in dealing with every level of Health worker. In October 1985, Mr Wally Mears was appointed to be Manager of the combined Mental Illness, Mental Handicap and Community Units and took up his post in January 1986. He comes from the Royal Air Force and a career as Navigator with, more recently, considerable experience of administration as a Group Captain and head of Air Staff at R.A.F. Upavon. He has a most difficult task in welding a number of large and disparate groups into a whole with an increasing emphasis on community based care.

We are pleased to see that the cream of the old administrative brigade have been incorporated into the new managerial support system at District Headquarters so that there is a feeling of community as we embark on the Sainsbury experiment. How commercial management techniques can be humanely applied to health care remains to be seen and clinicians will have a watchful eye on the expense/results ratio of such devices as management budgeting. We have not always warmed to change quickly, but we are all in favour of better treatment for more patients. We welcome our new managers and look forward to the first three years working with them for a better health service. We do not envy them the task of acting as arbiters where there is uncertainty or disagreement about priorities in health care. Neither do we envy them the pressure to perform under the discipline of a fixed term contract, with the danger that decisions could be made too hastily.

R. W. Hiles

### Ologists

With the increasing complexity of medicine and surgery and the development of new and complex treatments, conflicts between generalists and ologists are bound to occur. One is the basic problem of emergency admissions: many neurologists, haematologists, nephrologists, rheumatologists and cardiologists for example wish not to be involved in the emergency rota. It can be argued that this leaves them more free to develop their own speciality, to attend international meetings and so forth, being absolved from on call regularly, having to shoulder responsibility for seriously ill emergencies, and training students and juniors on such patients. Nor would it be sensible for all patients to be screened on admission, and all strokes, headaches, overdoses and fits diverted to neurologists; all with cough or dyspnoea going to a chest physician; those with belly ache being channelled to a gastro-enterologist, etc. Since four fifths of medical admissions to a general hospital are emergencies, this system would demand the services of an experienced doctor to act as a sorter, and all the ologists would need to be available all the time.

The model which is gaining favour for the average general hospital is to have generalists with special interest, and this is supported by the Royal College of Physicians. Thus most of the physicians take their turn on emergency rota, but develop their ologies mostly for non-urgent consultations in out-patients and in the beds of their colleagues. Most branches of medicine accept the need for this type of physician, though the neurologists still regard their discipline as 'pure'. I think pressure from time expired senior registrars and peer pressure will make them alter their view so that most districts in the land will have a neurological opinion available from their own staff. There will still be centres of excellence in association with neurosurgical units, or in the case of cardiology linked with cardiac surgery. The question then has to be asked: are such high powered centres, whether surgical or medical, the correct place for undergraduate teaching? I suggest that for the future students will get a better deal when taught in district general hospitals, and good general practices.

H. G. Mather

### 'What is Truth?'

Despite the immunogenic form in which most subjects were imparted during my school days, some things made a lasting impression. Among these were the essays of Francis Bacon, undoubtedly because of their pithy, quintessential opening statements. As I write it is his essay 'Of Truth' which comes to mind. 'What is truth?' said jesting Pilate; and would not stay for an answer! Why? Because the Westland affair is at its height the comedy and tragedy of which are more intriguing than any television soap opera.

The Times of January 16th is open in front of me and the editorial makes no bones about it. 'Someone is telling the truth and someone is not.' But, as Francis

Bacon reminds us, the problem is to determine—wherein the truth lies.

Although the future of Westland is important to us in the South-West, it is not a matter of global proportions. Both the rumpus and its reverbations are out of all proportion to the initial stimulus. At the heart of the matter is what this debacle tells us of Her Majesty's government's (yes, it is Her Majesty's and not Maggie Thatcher's!) overall approach to the making and implementation of policy.

I can see exactly the same problem writ large over the handling of the National Health Service. Government figures tell us that more is being spent on more doctors, more nurses and more services than ever before. But what is the perception of those of us working at the front line of patient care (in whatever capacity)? I find my own view to be shared by everyone to whom I speak—a worsening service, demoralised by continual reorganisation, unappreciative of the 'goodwill' of so many overstretched staff, and following well-meaning but cockeyed ideas emanating from the Elephant and Castle. These views are, surely, mutually exclusive. Wherein, then, lies the truth?

It is likely to be somewhere between these two extremes but since regional and district treasurers can show the calculations emanating from central government to be in error, one must take them with the proverbial pinch of salt. A colleague (Tory from the cradle) recently wrote to his Conservative MP giving facts and figures about clearly deteriorating patient care. A foolish reply berated him for 'believing and perpetuating Socialist propaganda' clearly implying that my colleague was lying. In the last analysis it seems that the truth itself is not the first priority—*it is more what the powers that be need to look like truth.* Image triumphs over veracity. Don't get me wrong, what the government says is *partly* true but as Francis Bacon has it 'mixture of falsehood is like alloy in coin of gold and silver, which may make the metal work the better, but it embaseth it'.

G. M. Stirratt

**They shoot horses, don't they?**

The nearest that this particular correspondent has ever got to a real live horse was the time when he mistakenly strayed into a race-course in the North of England. At the time he was proudly engaged to be married and was escorting his fiancée in foreign territory. His inability to actually place a bet on a horse without the help of half the police force, and the assistance of the other half of the book-keeping fraternity remains an embarrassing reminder that all is not well with the medical education provided in London teaching hospitals. Dublin apparently does much better. Irish fiancées must be much more reassured by the *savoir faire* of their future husbands.

Truth to tell, the only thing that I do know about horses is that you are meant to be able to tell their age from the state of their teeth. This horse, your correspondent, is ageing fast. Not so long ago a front tooth decided that enough was enough, and gaily left its former home at the prompting of a jam doughnut.

Until that moment I had an only vague recollection that consonants are divided into labials, fricatives and palatals. Clinical medicine is not just academic, although I should explain at the time that I was trying to do modest justice to academia. The real problem was that there was an examination on. Not merely an examination, but viva voces to conduct with no front tooth. Normally one thinks that it is the candidate who does the *voce* part of this exercise. Let me tell you, with painful experience that this impression is totally false.

Whilst thoughtfully and discreetly wrapping the errant tooth in a handkerchief, it was quickly obvious that there were a lot of words that it was just not possible to say to an apprehensive candidate. No one, least of all someone who is paying for the conversation, likes to hear 'th's', 'sh's' and 'f's' disappear in a struggle of muffled and hissing enunciation.

In a suddenly toothless state there is no possibility of asking direct questions about schistosomiasis, syphilis, foetal distress or cysticercosis. Neonate, tumour, metabolic need, and other 'palatal' ills are the order of the day.

I have two suggestions. The first is that books on examination technique for candidates should bear the acutely toothless examiner in mind. The second is that old horses need shooting if they can't ask about synapses, thalassaemia, or the thoracic thymus.

J. D. Davies

